# On the Capability of QoE Improvement Based on the Adjustment of RLC Parameters

**DOI:** 10.3390/s20092474

**Published:** 2020-04-27

**Authors:** Jessica Mendoza, Isabel de-la-Bandera, David Palacios, Ana Herrera-García, Raquel Barco

**Affiliations:** 1 Department of Communications Engineering, University of Malaga, 29071 Málaga, Spain; ibanderac@ic.uma.es (I.d.-l.-B.); ahg@ic.uma.es (A.H.-G.); rbm@ic.uma.es (R.B.); 2 Tupl Spain S.L., Tupl Inc., 29010 Málaga, Spain; david.palacios@tupl.com

**Keywords:** quality of experience (QoE), end-to-end (E2E) optimization, radio link control (RLC), mobile communications networks

## Abstract

The constant evolution in mobile communications networks have led operators to seek new techniques to optimize their mobile networks with the objective of satisfying the expectations of the users. In this way, traditional optimization techniques based on improving radio indicators, have given way to new techniques based on improving the quality of experience (QoE) perceived by users. This paper is focused on analyzing the impact of the adjustment of radio link control (RLC) layer configuration parameters on the QoE perceived by the users of two different types of services. Firstly, an evaluation of the QoE experienced by the user of a real-time video streaming service with respect to the transmission buffer size of the RLC layer in unacknowledged mode (UM) has been carried out. Secondly, the QoE perceived by the user of a file transfer service in relation to the variation of the configuration parameters of the RLC layer in acknowledged mode (AM) has been evaluated. The study, which has been carried out in a simulated cellular environment, has been performed for different system bandwidth values, thus proving the relationship between the QoE perceived by the users, the optimal RLC configuration parameters values and the available bandwidth.

## 1. Introduction

Mobile communications networks are constantly evolving. The increase in the number of subscribers, as well as the growth of traffic demand make the network increase, not only in size, but also in complexity. The introduction of new services that require more and more radio resources and the growth of users’ expectations have led mobile network operators (MNOs) to seek new techniques to optimize their networks.

The first attempts to optimize mobile networks were centered in optimizing a specific network segment, mainly, the radio access network (RAN), acting on key performance indicators (KPIs) of that segment, such as delay or call block rate (CBR) [1]. Over time, the purpose of the optimization process has been changing, increasingly approaching to users’ perception of the offered services. This change of focus in the goal of network optimization is accentuated with the arrival of the fifth generation (5G) networks [2]. In these networks, with the aim of providing the best possible user experience, a user-centric management approach is proposed [3]. As a result, traditional optimization techniques are being replaced by new techniques with a user-centric approach, which rely on user metrics to tune networks. The first solutions proposed with this philosophy were based on key quality indicators (KQIs). KQIs are a set of metrics that provide information about the service quality at application level, unlike KPIs, which only provide network-specific performance information. Some works, such as [4], present a study of various aspects of mobility enhancements in order to reduce the service interruption time. Currently, the research community is focusing on solutions to optimize the degree of final users’ satisfaction. These solutions are based on quality of experience (QoE) metrics. QoE measures quality from the point of view of user perception. Today, there are mathematical models that approximate the value of the QoE by computing different KQIs. The KQIs used to calculate the QoE can vary depending on factors such as the service being valued or the scenario in which the communication is taking place [5]. Some examples of KQIs used for calculating QoE metrics are: throughput, packet loss rate or waiting time. QoE metrics include the effect of the end-to-end (E2E) system and reflect the subjective quality perceived by users.

In the literature, different studies related to the optimization of the QoE perceived by users for different types of services can be found. Some authors address the optimization of the QoE from an application layer perspective. Thus, in papers such as [6,7], metrics as the video initial time, the receptor buffer state or the application bitrate are used to compute the QoE and perform the optimization of a video streaming service. In [8], to adapt the bitrate of a video streaming service in a dynamic way, an algorithm based on the QoE and the application layer buffer occupancy is proposed. However, other authors address the issue from lower layers perspective. Authors in [9] propose the use of new schedulers based on the QoE of real-time video streaming. In [10], the QoE of a third generation (3G) voice call service is optimized by adjusting the tilt of the antennas.

In wireless communications, as it is the case of mobile communications networks, the use of the radioelectric spectrum, as transmission medium between the sender and the receiver, causes the transmitted signals to be degraded due to interference, noise and fading. In mobile communications, the radio link control (RLC) layer is responsible for providing reliable communications between the transmitter and the receiver and reducing the impact of these phenomena. RLC provides a reliable wireless link, performing error detection and, in some cases, correction tasks. Depending on different service requirements, the use of error detection and correction tasks may be more or less adequate. For instance, in services such as file transfer or web browsing, due to its strict reliability requirements, it will be suitable to retransmit lost packets; however, in real-time services, such as video calls, to meet the low-latency requirement, the use of retransmissions will not be appropriate. The optimization of the RLC layer is an issue dealt in articles such as [11], where an analysis of the operation of RLC layer in acknowledged mode (AM) in Universal Mobile Telecommunications System (UMTS) technology is made, or in [12], where a preliminary analysis of the performance of the user datagram protocol (UDP) and transmission control protocol (TCP) on the different operating modes of RLC, AM and unacknowledged mode (UM) is conducted. However, in [9], the impact on users’ experience due to the adjustment of RLC parameters is not analyzed, and in [12], the configuration of the different RLC modes is not studied in detail; instead, the work in [10] focuses on the improvements that one mode provides over another.

The aim of this work is to analyze the impact of the adjustment of RLC parameters on the QoE perceived by users. Specifically, two services have been selected: real-time video streaming and file transfer. This work extends the analysis presented in [11]: firstly, including the evaluation of the operation of the RLC layer in UM mode and extending the analysis of the operation of RLC AM including the study of new timers. Secondly, considering RLC in the context of a Long-Term Evolution (LTE) RAN. Thirdly, analyzing the effect of this layer on the QoE perceived by the user.

Although this analysis is focused on LTE, due to the great similarity of the RLC layer in LTE and 5G technologies, the presented study could be easily reproduced in a 5G network. Thus, obtaining conclusions on QoE improvements in 5G environments due to the adjustment of the RLC parameters.

## 2. Radio Link Control

RLC is a layer-2 sublayer, situated between packet data convergence protocol (PDCP) layer and the media access control (MAC) layer [13] and is controlled by the radio resource control (RRC) protocol. The main functions of RLC are the segmentation and reassembly of RLC service data units (SDUs), as well as error detection and correction along the radio interface. The RLC layer can be configured in three modes:

1. Transparent mode (TM). TM does not consider the segmentation and reassembly of the packets before delivering them to adjacent layers. This mode is used to exchange control messages with the RRC layer.
2. Unacknowledged mode. TM and UM RLC modes do not support the retransmission of lost packets. Thus, these modes are considered as unidirectional communication modes. Contrary to TM mode, UM does the segmentation and reassembly of packets. The functionalities of the RLC layer in UM mode are executed by two entities: a transmitting UM RLC entity and a receiving UM RLC entity. The transmitting UM RLC entity receives packets from the PDCP layer and stores them in a transmission buffer, waiting for a transmission opportunity notification from the MAC layer. The receiving UM RLC entity receives packets from the MAC layer and stores them in a reception buffer. To manage the information flow between RLC and PDCP layers, a timer called t-Reordering is used. When the t-Reordering timer expires, the receiving UM RLC entity reorders the packets and delivers them to the PDCP layer. UM is normally used when delay takes precedence over reliability. That is the case of real-time services such as voice calls or video streaming.
3. Acknowledged mode. AM is characterized by allowing the retransmission of lost packets, establishing a bidirectional communication between the transmitter and the receiver. Furthermore, this mode does the segmentation and the reassembly of the packets. To establish an RLC communication in AM mode, an AM RLC entity is needed both in the transmitter and in the receiver. An AM RLC entity consists of a transmitting side and a receiving side. In addition to perform the same functions as the RLC UM entities, RLC AM entity support lost packets retransmission. To control the flow of packets between the transmitter and the receiver, RLC AM uses both a transmitting and a receiving window. The transmitting window is formed by the set of packets sent by the transmitter from which a confirmation message was not received. The receiving window delimits the range of packets that are allowed to be received. If the receiving side detects lost packets, it may request retransmissions of the packets to its peer AM RLC entity. To this end, the receiver sends a STATUS PDU to the transmitter. The STATUS PDU contains positive and/or negative acknowledgment (ACK and/or NACK) of the received RLC PDUs. STATUS PDUs are sent on request of the transmitter. To control the number of STATUS PDU requests the transmitter implements the following parameters:
  - pollPDU and pollByte: pollPDU indicates how many PDUs the transmitter will send before requesting a STATUS PDU. In the same way, pollByte indicates how many bytes the transmitter will send before requesting a STATUS PDU. When one of these limits is achieved, the transmitter includes a poll in the next packet sent to the receiver.
  - t-PollRetransmit: this timer determines the transmitter waiting time before retransmitting a STATUS PDU request. When the transmitter sends a packet with a poll, the t-PollRetransmit is started. If a STATUS PDU is received while the timer is running, the transmitter stops and resets the timer. If the timer expires and no STATUS PDU has been received, the transmitter includes a poll in the next packet sent to the receiver. This packet could be, in case the transmitting window is full, a retransmission.
  - maxRetxThreshold: this parameter limits the number of allowed retransmissions for a packet.
  To control the frequency of STATUS PDU transmissions the receiver implements the following timers:
  - t-StatusProhibit: it indicates the receiver waiting time between sending two consecutive packets.
  - t-Reordering: as in the UM case, when the t-Reordering timer expires, the receiving AM RLC entity reorders the packets and delivers them to the PDCP layer, detecting lost RLC PDUs.
  AM is used for services in which reliability often takes precedence over delay, such as web browsing or file transfer.

## 3. Services

In this section, a brief summary of the services that have been used in this work is presented, as well as a measurement of their corresponding user-perceived QoE. These services are real-time video streaming and file transfer by file transfer protocol (FTP).

### 3.1. Real Time Video Streaming

Nowadays, most of the Internet protocol (IP) traffic going through mobile networks is due to video services. According to studies carried out by CISCO [14], in 2021 these types of services will constitute the 82% of all Internet traffic, that implies, in mobile networks case, a 55% increase in the consumption of these services.

The QoE is defined as the users’ perception of the received service quality. The most widespread way to quantify the QoE in video streaming services is the use of the mean opinion score (MOS) scale. The MOS scale evaluates the QoE perceived by users assigning it a value which can range from 1 (bad) to 5 (excellent). Since the MOS is a user perception metric, this is computed subjectively, making tests to groups of users in controlled environments. However, subjectively calculating MOS presents two main problems, on the one hand that it is a slow method, and on the other hand that it is expensive. To solve these problems, several authors have focused their studies on calculating empirical models that serve to make an effective estimation of the MOS. These models are based on the use of performance measures of networks and/or services. The MOS estimated using mathematical models is known as objective MOS. In this paper, an objective MOS model is used.

Regarding video services, the most popular performance metrics used to approximate the MOS value are the peak signal to noise ratio (PSNR) and the structural similarity (SSIM), which measure the received video quality degradation. To that end, a comparison between the video before the transmission and the received video is performed. However, the MOS models considering these metrics have some limitations, since they do not consider aspects like the frame loss during the transmission.

The QoE in this work has been measured using an alternative method to compute the objective MOS, defined in [15]. This method considers the packet loss rate, as well as the degradation of the frames during the transmission, thus obtaining a better approach to the subjective MOS. This model of MOS achieves a Pearson correlation of 0.9509 with the subjective MOS, which is a high correlation value, demonstrating that the model is true to reality.

(1) $$MOS=4.367-0.5040\frac{d}{dPSNR}-0.0517l$$

where *d* is the distorted frame rate, *dPSNR*, the average PSNR for all distorted frames and *l*, the frame loss rate. In this work, a frame is considered as degraded when the value of the PSNR of this one is lower than the 5th percentile of the video frames PSNR being sent before.

### 3.2. File Transfer Service

FTP is a typical file download service. To evaluate the QoE experienced by users in FTP services, metrics as the download duration, the mean E2E throughput or the MOS are normally used. Different authors propose different definitions of the objective MOS for FTP services. These definitions have in common that they depend on throughput perceived by clients [16]. Specifically, there are studies such as [17] where authors used the Weber–Fechner Law (WFL) of psychology of perception to demonstrate that the QoE in file transfer service has a logarithmic relationship with the throughput reached in the transmission and, in the same way, with the time it takes to download the file. To give a global vision and not restricted to the definition of a particular objective MOS, in this paper the E2E throughput is directly used as an indicator of the QoE of an FTP service.

## 4. Impact of RLC Layer Configuration

Taking into account the operation of the RLC layer in AM and UM modes and the main requirements of the two types of services studied in this work, the following premises can be made.

Real-time video services are characterized by having strict requirements in terms of latency. These types of services often use the RLC UM mode, as specified above. The RLC UM mode does not support retransmissions, allowing applications that run over it to reach low delays. In return, these applications experience an increase in the packet loss rate. Despite having flexible requirements in terms of packet loss, a high packet loss rate may cause a quality degradation in the received video, and therefore a deterioration in the quality perceived by users.

In the RLC UM transmission buffer, two phenomena that cause losses and degradation of the transmitted service may appear. These phenomena are: the discard of packets due to the lack of available space in the buffer and the delays produced by the accumulation of packets in the buffer.

If the available bandwidth in the system is big enough to cover the traffic demand coming from the server, the packets will be sent to the receiver and the accumulation of the packets in the RLC transition buffer will not be produced. Thus, if the packet delivery rate is higher than the packet generation rate, the losses and the delays produced in this layer are null. By contrast, if the bandwidth available in the system is too small, the accumulation of the packets in the RLC buffer will be so high that this will be continuously collapsed, producing a high packet loss rate. Therefore, to appreciate the effect of the RLC UM buffer size optimization it will be necessary to find a suitable scenario. This scenario will depend on the radio channel conditions and the quality of the service to transmit.

Unlike real-time video services, file transfer services are characterized by strict requirements in packet loss rate, being more flexible with the delay produced during the file transmission. Therefore, file transfer services often run over the RLC AM mode. To perform the error correction (for the retransmission of wrong and lost packets) the RLC AM mode uses the automatic repeat request (ARQ) algorithm, minimizing in this way the packet loss rate. However, a misconfiguration in the ARQ algorithm may lead to an excessive latency and, therefore, to a poor QoE.

The transmitting side of the RLC AM entity prioritizes the transmissions of RLC control PDUs over RLC data PDUs. At the same time, retransmissions of RLC data PDUs have priority over transmission of new RLC data PDUs. Thus, a frequent delivery of control packets (STATUS PDU) and retransmitted AMD PDUs will cause further delay when sending new data packets.

The delivery frequency of control packets depends on the configuration of the RLC AM transmitter and receiver parameters. If the pollPDU and pollByte parameters are configured with small values, the transmitter will continuously request STATUS PDUs. If the pollPDU and pollByte parameters are configured with large values and the timer t-PollRetransmit is configured with a small value, the situation will be similar to the previous one. In some cases, a small value of t-PollRetransmit may lead to the delivery of unnecessary retransmissions. In the receiver side, the timer t-StatusProhibit controls how often a STATUS PDU is sent to the transmitter. If the t-StatusProhibit has a small value, the transmitter request will be answered almost immediately, producing, in the case of small values of transmitter parameters (pollPDU, pollByte and t-PollRetransmit), the delivery of many control packets, which introduces delays in the data transfer. If the t-StatusProhibit or the transmitter parameters (pollPDU, pollByte and t-PollRetransmit) have large values, it will take a long time to receive the ACKs of the delivered packets, producing the overflow of the transmitting window. As a result, detection and correction of error tasks will take more time, producing in higher layers (mainly TCP) the delivery of retransmissions, that degrades the QoE. When the number of retransmissions of a packet reaches the maxRetxThreshold, the packet is discarded. The update period of the state variables (ACKs and NACKs) that are sent in the STATUS PDU depends on the value of the t-Reordering timer.

## 5. Performance Analysis

In this work, different tests to confirm the recommendations on configuration parameters previously stated have been carried out by simulating an LTE mobile network. Firstly, a study to analyze the impact of the adjustment of the RLC UM transmission buffer size on the QoE perceived by users of a real-time video streaming service has been performed. Secondly, a study to analyze the impact of the adjustment of the RLC AM parameter on the QoE perceived by users of a file transfer service has been carried out. The LTE module of the NS3 simulator, called LENA [18], has been used to carry out these tests.

The simulation scenario consists of two LTE cells, each one with 10 users, although only one of them is analyzed. The scenario includes mobility as well as random initial positions of users. The user being assessed is the client of a real-time video streaming service in the first case, and of a file transfer service in the second case. The rest of the users are configured as clients of a constant bitrate service in both cases. This bitrate is the same for the tests performed with different bandwidth values. Thus, the network resource availability is very low for small bandwidth values, and high, for large bandwidth values. The video transmitted by the video streaming server is characterized by having a MPEG-4 format, a size of 3.6 MB and a duration of 10 s. The video has a frame rate of 30 fps. To meet the real-time video streaming services, those packets that experienced an E2E delay greater than 150 ms will be considered as lost. File transfer tests are executed with 5 MB files. The main configuration parameters of the simulations are shown in Table 1.

The reliability of the results is guaranteed thanks to the use of mobility on the scenario. The QoE values provided correspond to the average QoE value during the simulation, reflecting the QoE perceived by the studied user during the different user positioning situations that occur during the simulation. In this way, the results obtained are representative of the entire scenario.

### 5.1. Evaluation of Real-Time Video Streaming Service

The traffic of the real-time video streaming service has been generated and subsequently assessed with the Evalvid tool [19]. Evalvid, in addiction to typical metrics of video quality such as the PSNR, provides KPIs related to the subjacent network, such as loss rate, delay or jitter.

**Figure 1:**
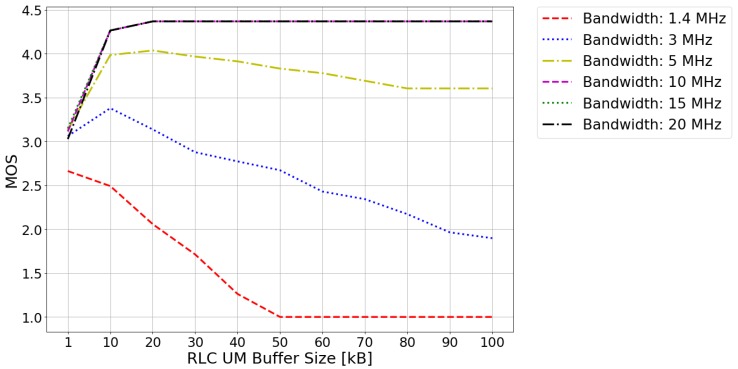
Expected mean opinion score (MOS) for different system bandwidths, as a function of the radio link control (RLC) unacknowledged mode (UM) buffer size.

Figure 1 shows the results of MOS obtained for different RLC UM buffer sizes and system bandwidth values. As can be appreciated in the figure, the MOS can be improved by increasing the bandwidth available in the system. This is because, the more bandwidth available in the system, the more data can be transmitted by the video server in each TTI, achieving a higher transmission speed and therefore a better QoE.

Two different behaviors can be observed in the curves depicted in Figure 1. The first case occurs when the server packet generation rate is higher than the packet transmission rate at the MAC level. The curves obtained for bandwidth values of 1.4, 3 and 5 MHz present this behavior. The second case occurs when the packet generation rate is similar to the packet transmission rate. This behavior can be appreciated for bandwidth values of 10, 15 and 20 MHz. For each bandwidth value, an optimum RLC UM buffer size that maximizes the MOS has been found. This value is the result of a tradeoff between the losses caused by a too small buffer size and the delays caused by a too large buffer size. On the one hand, in too small buffer size cases, having a little storage space for the packets received from the PDCP layer, the RLC UM transmission buffer will quickly collapse, causing packet loss. On the other hand, in too large buffer cases, the accumulation of packets in the buffer may cause the maximum established end-to-end delay restriction (150 ms) to be exceeded. However, depending on the case, the behavior of the curve after the optimal result is different. For the first case, the MOS value will decrease as the buffer size increases. This is because when the size of the buffer exceeds the optimum, the packets that are generated will begin to accumulate in the buffer, resulting in greater delays and increased losses. In the second case, for a certain buffer size value the MOS value remains constant. This steady behavior is due to the fact that, for a certain buffer size value, the buffer occupancy is always the same, meaning a balance between the packet generation rate and the packet transmission rate. For bandwidth values of 10, 15 and 20 MHz, the buffer size value from which the MOS remains constant matches with the optimum buffer size value. This is due to the fact that, in the simulated scenario, the delays caused by the accumulation of packets in the buffer are not large enough to exceed the E2E limit delay.

As a result, it can be inferred that the value of the RLC UM transmission buffer size, with which the maximum QoE is experimented by the user, depends on the relation between the server packet generation rate and the packet transmission rate and, therefore, on the system available bandwidth. In the scenario studied in this paper, in 1.4 MHz system available bandwidth case, the optimum RLC UM transmission buffer size is 1 kB; in 3 MHz, 10 kB; and from 5 MHz system available bandwidth onwards, 20 kB.

### 5.2. Evaluation of File Transfer Service

To perform the QoE optimization of a file transfer service, two of the RLC AM parameters has been used. The RLC AM parameters selected to perform the optimization tasks have been the t-StatusProhibit and the t-PollRetransmit, since, based on the operation of the RLC AM mode, these two parameters seem to have a great effect on the E2E throughput.

**Figure 2:**
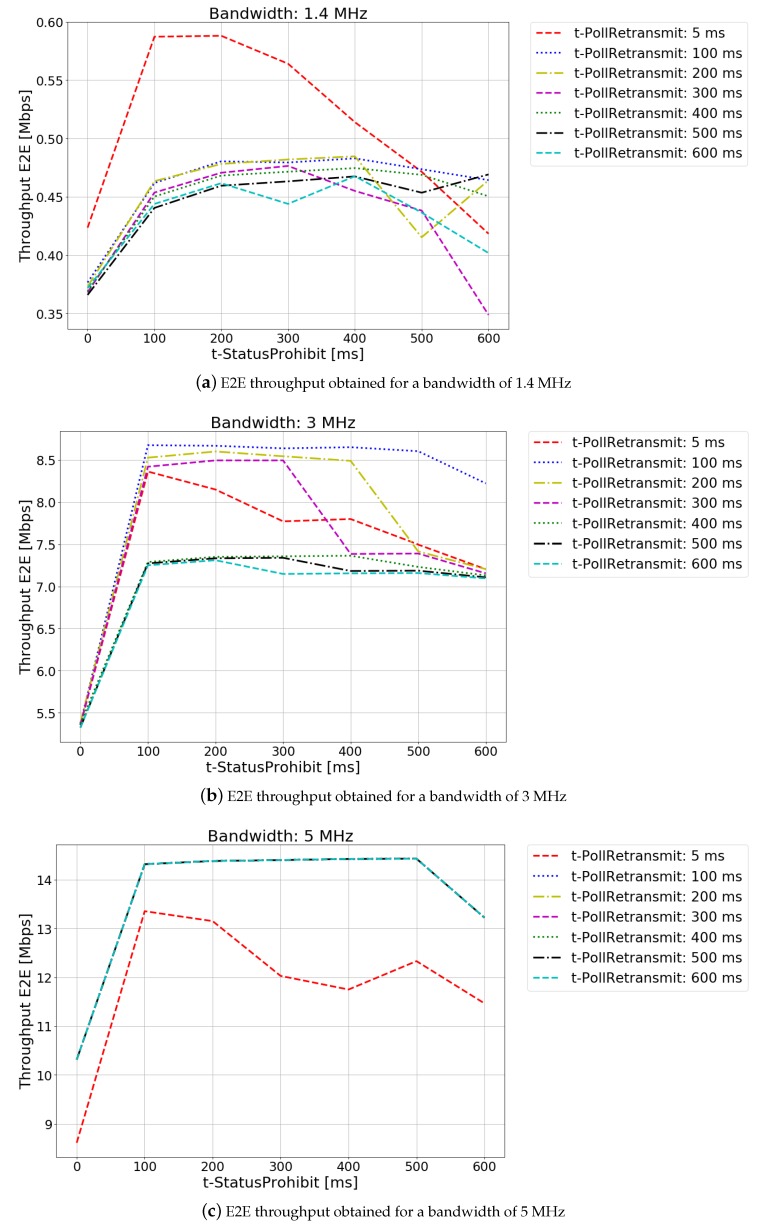
End-to-end (E2E) throughput optimization.

In Figure 2, the results of the optimization are shown. Each subfigure represents the study performed for system bandwidths of 1.4, 3 and 5 MHz, respectively. The studies for larger bandwidths are not shown since, in this scenario, the behavior is the same as for a bandwidth of 5 MHz.

Comparing the different subfigures that make up Figure 2, it can be seen how the greater the bandwidth available in the system, the greater the E2E throughput perceived by the users. This is due to the fact that, by increasing the bandwidth while maintaining the same number of users in the cell, users will have a greater amount of resources to transmit or receive.

For system bandwidths of 1.4 MHz and 3 MHz (Figure 2a,b), it can be appreciated how the E2E throughput varies with both t-StatusProhibit and t-PollRetransmit. For small values of t-StatusProhibit and t-PollRetransmit the E2E throughput experienced by users decreases. This is due to the excess of control packets (STATUS PDUs) sent by the receiver to the transmitter. As it is mentioned before, the RLC AM mode prioritizes the delivery of control packets over data packets, producing a throughput degradation. For high values of t-StatusProhibit and t-PollRetransmit the E2E throughput perceived by users decreases too. In this situation, the STATUS PDUs will be sent infrequently. As a result, the transmission window will have a slow progress, and may eventually collapse, producing the delivery of unnecessary retransmissions.

In the cases with the highest bandwidth values, where the amount of available resources is sufficient to absorb the traffic demanded by users, the effect produced by the variation of the t-PollRetransmit disappears for timer values higher than 100 ms (Figure 2c). This is because the network has sufficient resources to absorb the traffic generated by the FTP server, keeping the transmitter transmission and retransmission buffers empty. When the transmission and retransmission buffers are empty, a PDU with the poll bit set to 1 is sent. As a result, STATUS PDUs requests are sent before the t-PollRetransmit timer expires. The effect caused by the t-StatusProhibit timer remains the same. For small values, when there is an oversupply of control packets, the value of the E2E throughput decreases. For large values of the t-StatusProhibit timer, the number of retransmissions increases due to the lack of confirmation of the received packets.

For the same service, depending on the available system bandwidth, the t-PollRetransmit and t-StatusProhibit timers with which the maximum value of E2E throughput is achieved will change. Since both the t-PollRetransmit and t-SatatusProhibit timers affect in the progress of the RLC AM transmission window, these timers have an impact on the E2E throughput experienced by the user. In the scenario studied in this paper, in the 1.4 MHz system available bandwidth case, the optimum values are between 100 and 200 ms for the t-StatusProhibit timer and 5 ms for the t-PollRetransmit timer; in the 3 MHz system available bandwidth case, the optimum value of t-StatusProhibit is between 100 ms and 400 ms and the optimum value for t-PollRetransmit is 100 ms; and for cases of 5 MHz system available bandwidth and above, the optimum value for t-StatusProhibit is 500 ms and the optimum values for t-PollRetransmit range from 100 ms onwards.

## 6. Final Recommendations

To sum up, final recommendations are presented in this section, based on the results described in the previous section. These recommendations depend on the environment conditions, such as the system available bandwidth or the type of service to be addressed.

From the results, it can be inferred that the use of an optimization algorithm based on the adjustment of the RLC layer parameters would allow to improve the QoE experienced by users adapting this configuration to the specific conditions of a certain scenario. Regarding the optimization techniques that could be used to implement the optimization algorithm, it is possible to distinguish between two groups: model-based techniques and control-based techniques. On the one hand, the first group of techniques guarantee the optimality of the algorithm, however, it is necessary to have a very high precision network model to implement this kind of methods. On the other hand, control-based techniques do not need any network model. In this kind of techniques, different network configurations will be tested, and depending on the results, the next steps will be decided. This kind of techniques has been widely used in mobile networks optimization.

Below, there are some recommendations to define the inputs of a QoE optimization algorithm by the adjustment of RLC parameters, based on control techniques:

- First, it is important to feed the algorithm with the information about the type of service requested by users in order to select the proper RLC mode, AM or UM.
- Depending on the selected RLC mode, the optimization algorithm should know the current value of the RLC UM transmission buffer size, in the case of UM mode, and the values of t-PollRetransmit and t-StatusProhibit RLC AM timers, in the case of AM mode.
- Finally, the algorithm should be feed with the current value of QoE experienced by the user.

The optimization algorithm should return the value of the RLC UM transmission buffer size, in the case of UM mode, and the values of t-PollRetransmit and t-StatusProhibit RLC AM timers, in the case of AM mode, that maximize the QoE perceived by the user. Finally, Table 2 shows the RLC layer recommended configuration for each of the studied use cases.

## 7. Conclusions

In this paper, an analysis to evaluate the impact of the adjustment of RLC parameters on the QoE perceived by users is presented. The analysis has been performed in a LTE environment, however, due to the similarity between the RLC layer in LTE and 5G technologies, the analysis could be easily reproduced in a 5G scenario. In this sense, although the results obtained in this paper are not directly applicable to 5G, the proposed methodology is valid for improving the QoE of users of 5G networks.

Firstly, using a real-time video streaming service, the QoE perceived by the users has been evaluated with respect to the RLC UM transmission buffer size variation. The RLC UM transmission buffer size directly affects losses and delays suffered by the sent packets. The evaluation has been performed for different system bandwidth values, thus proving that the optimal value of the RLC buffer size and the maximum QoE experienced by the users are related to the available bandwidth. This behavior is due to the amount of resources assigned to the user in each moment. The higher the bandwidth, the larger the optimum RLC UM transmission buffer size and the greater the QoE perceived by the users.

Secondly, the evaluation of the QoE experienced by the users of an FTP service with respect to the variation of the RLC AM timers t-PollRetransmit and t-StatusProhibit has been carried out. These timers affect the delay experienced by the packets during the transmission. As in the previous case, the evaluation has been performed for different system bandwidth values, proving that these timers will have a greater effect when the amount of radio resources allocated to the user is limited.

Finally, it can be concluded that it is possible to optimize the QoE perceived by users by adjusting RLC layer parameters. Some recommendations are provided based on the obtained results. The development of an automatic optimization algorithm will be addressed in a future work.

## Figures and Tables

**Table 1:** Simulation scenario and main configuration parameters.

	Real-Time Video Streaming Service	File Transfer Service
**Scenario**	2 LTE cells	
	10 users in each cell	
	Distance between eNBs: 100 m	
**System Bandwidth**	1.4, 3, 5, 10, 15 and 20 MHz	
**RLC Mode**	UM	AM
	Transmission buffer size:	t-StatusProhibit: 0–600 ms
	1–100 kB	t-PollRetransmit: 5–600 ms
**Mobility**	Speed: 1.5 m/s Direction: Random	
**Transmission Direction**	Download	
**Services**	Studied user:	Studied user:
	Real-time video streaming service	File transfer service (file 5 MB)
	Other users:	Other users:
	Constant bitrate	Constant bitrate
**e-NodeB**	Omnidirectional antennas, SISO, EIRP ${}_{max}$= 30 dBm	
**Scheduler**	Proportional Fair	
**Transmission Time Interval (TTI)**	1 ms	
**Maximum Delay Allowed**	150 ms	-
**in Real-time Video Service**		

**Table 2:** Results summary.

**Use Case**	Real-time video streaming			
**RLC Mode**	RLC UM			
**Associated Configuration Parameter**	RLC transmission buffer			
**Optimization Tradeoff Parameter**	Excessive buffer: increased losses due to increasing delay			
	Insufficient buffer: increased losses due to increasing buffer overload			
**Recommended Values/Ranges**	Bandwidth (MHz)		Buffer size (kB)	
	1.4		1	
	3		10	
	5		20	
	10		20	
	15		20	
	20		20	
**Use Case**	File transfer			
**RLC Mode**	RLC AM			
**Associated Configuration Parameter**	t-PollRetransmit, t-StatusProhibit			
**Optimization Tradeoff Parameter**	Small values of the two timers: decreased throughput due to excess of control packets			
	High values of the two timers: decreased throughput due to the lack of control packets			
**Recommended Values/Ranges**	Bandwidth (MHz)	t-PollRetransmit (ms)		t-StatusProhibit (ms)
	1.4	5		100–200
	3	100		100–400
	5	100–600		500

## Abbreviations

The following abbreviations are used in this manuscript:

3G	Third generation
5G	Fifth generation
ACK	Acknowledgment
AM	Acknowledged mode
ARQ	Automatic repeat request
CBR	Call block rate
E2E	End-to-end
FTP	File transfer protocol
IP	Internet protocol
KPIs	Key performance indicators
KQIs	Key quality indicators
LTE	Long-Term Evolution
MAC	Media access control
MNOs	Mobile network operators
MOS	Mean opinion score
NACK	Negative acknowledgment
PDCP	Packet data convergence protocol
PSNR	Peak signal to noise ratio
QoE	Quality of experience
TCP	Transmission control protocol
TM	Transparent mode
TTI	Transmission time interval
RAN	Radio access network
RLC	Radio link control
RRC	Radio resource control
SDUs	Service data units
SSIM	Structural similarity
UDP	User datagram protocol
UM	Unacknowledged mode
UMTS	Universal Mobile Telecommunications System
WFL	Weber-Fechner Law
